# Use of ivabradine in a critically ill pediatric patient with recalcitrant ectopic atrial tachycardia and complex congenital heart disease

**DOI:** 10.1016/j.hrcr.2022.03.011

**Published:** 2022-03-18

**Authors:** Gretchen Hackett, Tracie K. Lin, Jason R. Imundo

**Affiliations:** ∗ Penn State Hershey Children’s Hospital, Hershey, Pennsylvania

**Keywords:** Automatic tachycardia, Pediatrics, Congenital heart disease, Ivabradine, Bradycardia

## Introduction

Ivabradine is the only hyperpolarization-activated cyclic nucleotide-gated (HCN) channel inhibitor currently approved for use in the United States. It decreases heart rate with minimal effect on blood pressure or inotropy, making it an attractive agent with an overall low side effect profile. The use of ivabradine in the pediatric population is limited, with current approvals only for dilated cardiomyopathy patients.^1^ Recent literature has supported the use of ivabradine in neonatal patients with junctional ectopic tachycardia.^2^, ^3^, ^4^, ^5^, ^6^ Ectopic atrial tachycardia (EAT) is an automatic tachycardia that can be seen postoperatively in patients after surgery for congenital heart disease (CHD).^7^^,^^8^ These patients have an increased risk of EAT when there is atrial stretch secondary heart failure and/or significant atrioventricular (AV) valve regurgitation. Owing to the incessant nature of EAT combined with the baseline tenuous hemodynamic status of a postoperative CHD patient, there is concern for morbidity and mortality if faced with this clinical scenario. Many of the currently used antiarrhythmic agents for EAT have negative effects on cardiac hemodynamics, potential for toxicity, and increased risk for arrhythmia.^4^ Ivabradine represents a potential alternative therapy for automatic tachycardia in tenuous pediatric postoperative CHD patients, which may provide a more hemodynamically favorable means of controlling postoperative EAT.

## Case report

**Figure 1:**
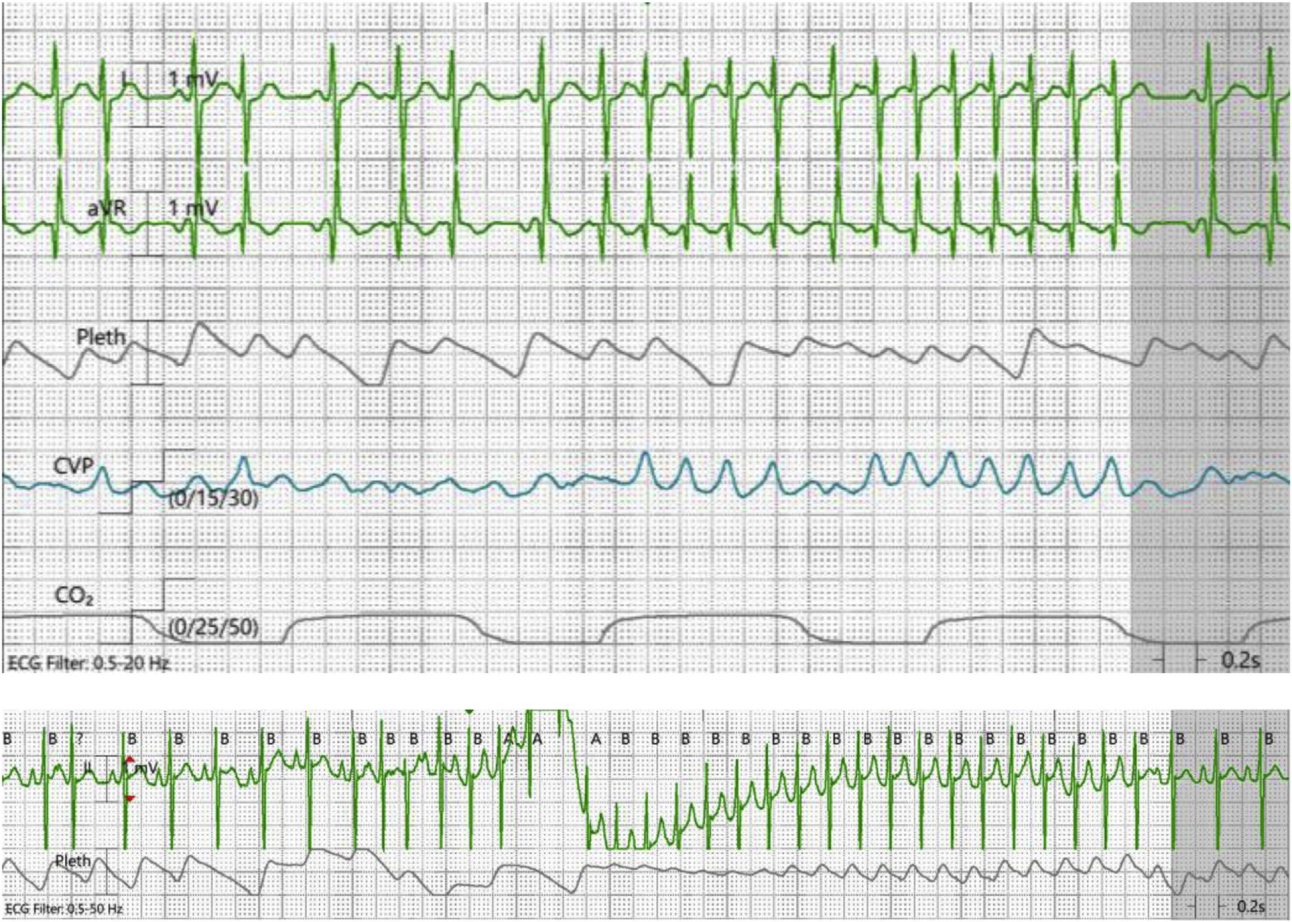
Runs of atrial ectopy. Plethysmography pulsatility changes between sinus beats and ectopic atrial tachycardia are appreciated.

Our patient was born at 37 6/7 weeks’ gestation with hypoplastic left heart syndrome (mitral and aortic atresia) and severe tricuspid valve regurgitation owing to incomplete coaptation of the tricuspid valve leaflets. Her birth weight was 3.3 kg. She underwent stage 1 palliation with Norwood procedure and Sano shunt at 4 days of life. She developed multifocal EAT within the first days of life and was initially loaded with digoxin 16 mcg/kg the day prior to Norwood-Sano. No pacing wires were placed at the time of her Norwood procedure. Postoperatively, she was continued on parenteral digoxin 3 mcg/kg twice daily. She had breakthrough runs of EAT with rates up to 220 beats per minute (bpm), resulting in hemodynamic instability (Figure 1), prompting discontinuation of digoxin and initiation of sotalol adjusted for age factor nomogram to 12 mg/m^2^/dose every 8 hours on postoperative day (POD) 7. She continued with hemodynamically compromising periods of EAT, prompting discontinuation of sotalol and initiation of an amiodarone infusion at 7 mcg/kg/min on POD 10. She continued to have breakthrough EAT requiring as-needed amiodarone boluses, and enteral propranolol at 1 mg/kg/day divided every 6 hours was started on POD 11, which lowered baseline heart rates from the 130–150 bpm range to 110–120 bpm range. The amiodarone infusion was subsequently reduced to 3.5 mcg/kg/min on POD 12 for continued maintenance dosing. By POD 15, she was noted to have profound hypoxic and bradycardic events during ventilator weaning trials. Although acute hypoxemia appeared to be the proximate cause of episodic heart rate decreases, propranolol lowered her baseline heart rate such that further bradycardia was poorly tolerated from a hemodynamic perspective. As such, propranolol was discontinued.

At POD 18 she developed refractory EAT, which did not respond to 10 (1 mg/kg) boluses of amiodarone, nor to an increase in amiodarone infusion to 7.5 mcg/kg/min. An esmolol infusion was not tolerated owing to hypotension. She became hemodynamically unstable, with mean arterial pressures as low as 20 mm Hg during EAT. In response, a procainamide bolus at 15 mg/kg was given, with resultant conversion to normal sinus rhythm; a procainamide infusion was started at 40 mcg/kg/min; and amiodarone was discontinued. The procainamide drip was titrated to serum procainamide concentrations of 10–12 mg/mL and N-acetyl procainamide (NAPA) concentrations of 10–13 mg/mL. Serial electrocardiograms were followed and QRS and QTc duration remained <20% prolonged compared to her baseline. While on the procainamide drip, she had initial rhythm control, and intermittent bouts of EAT were addressed with adjustments to the procainamide infusion and trials of esmolol infusion. However, as before, esmolol use was limited owing to associated hypotension with escalation of the medication. Frequent electrocardiograms were obtained throughout this time to monitor the QTc interval, given overlap in use of several antiarrhythmics with no significant QTc prolongation. She had a period of clinical stability on this regimen, but on POD 37 had recurrence of EAT, requiring initiation of an esmolol infusion up to 250 mcg/kg/min.

**Figure 2:**
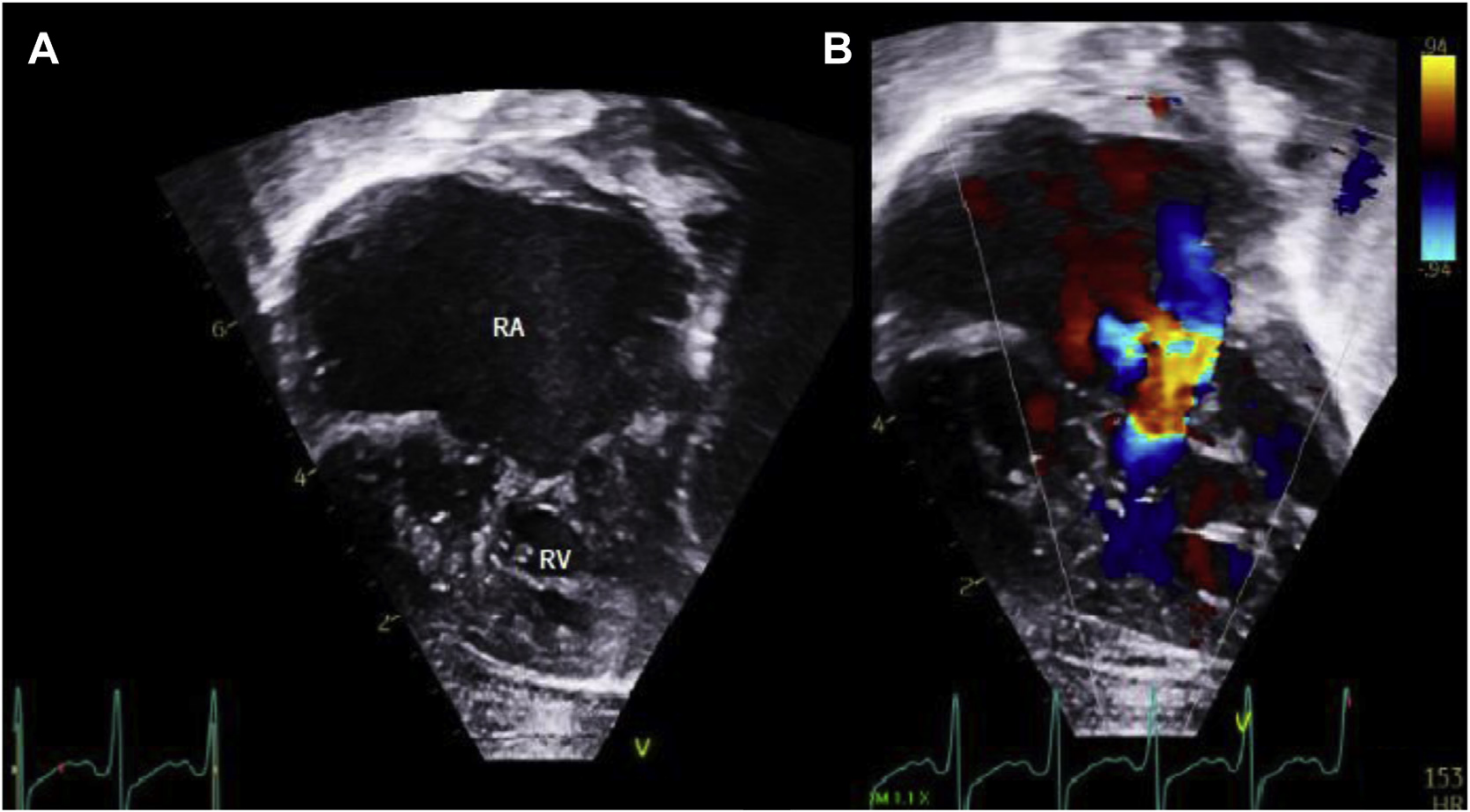
Echocardiogram demonstrating severe right atrial enlargement. A: Two-dimensional apical 4-chamber view demonstrating anatomy consistent with hypoplastic left heart syndrome. There is severe dilation of the right atrium. B: Apical 4-chamber view with color Doppler and severe tricuspid valve insufficiency owing to incomplete coaptation of valve leaflets. The severe tricuspid regurgitation led to severe right atrial dilatation, believed to be the nidus for incessant ectopic atrial tachycardia as well as heart failure. RA = right atrium; RV= right ventricle.

Owing to the severity of her tricuspid valve regurgitation with resultant severe right atrial dilatation as a likely contributing mechanism for her incessant EAT in conjunction with worsening heart failure, it was recommended she undergo tricuspid valve repair (Figure 2). This was done via anteroseptal commissuroplasty and posterior leaflet obliteration suture annuloplasty at 7 weeks of life and weight of 4.58 kg. She was brought back to our cardiac intensive care unit with an open sternotomy. Temporary atrial pacing wires were placed at the time of this surgery. The tricuspid valve repair resulted in improvement in her tricuspid regurgitation to mild-to-moderate and a mild increase in her tricuspid valve inflow gradients to 3 mm Hg. In the immediate postoperative period, she was noted to have frequent atrial ectopy, including runs of EAT. In the setting of overall hemodynamic instability and low cardiac output state following surgical intervention, the decision was made to initiate ivabradine at 0.1 mg/kg/dose twice a day, with subsequent increase to 0.1 mg/kg/dose 3 times a day 24 hours after initiation. For each ivabradine administration, a 5 mg tablet of ivabradine was crushed and mixed with 5 mL water, then 0.5 mL was administered to the patient via nasogastric tube. The remaining solution was discarded after each administration. The procainamide infusion was adjusted per electrocardiogram assessment and drug levels. On the increased dose of ivabradine there was quiescence of the EAT as the patient recovered from her tricuspid valve repair. During the initial period of recovery from the tricuspid valve repair with open chest physiology, all additional antiarrhythmics were able to be weaned off and EAT was controlled on ivabradine alone. Four days following the initiation of ivabradine, she began having clinically significant sinus bradycardia with resting sinus rates in the 80s–100s owing to chronotropic insufficiency. Associated clinical evidence of insufficient end-organ perfusion was noted. Atrial pacing via temporary epicardial wires was initiated, leading to subsequent improvement in cardiac output as manifested by markers of end-organ perfusion.

**Figure 3:**
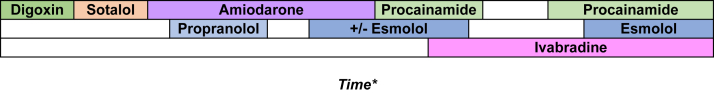
Timeline of antiarrhythmic regimens used in the care of our patient. ∗Not to scale. See case description for details of her antiarrhythmic medication administration.

Despite improvement in her arrhythmia management, with chest closure her overall care became complicated by challenging hemodynamics owing to AV valve regurgitation and elevated filling pressures. She developed chylous ascites, resultant fluid and electrolyte derangements, protein loss, coagulopathy, hypogammaglobulinemia, and occlusion of the superior vena cava. Transient episodes of breakthrough atrial ectopy in the form of isolated premature atrial contractions and runs of EAT occurred. The ivabradine alone could not control the atrial arrhythmias with the added hemodynamic stress. Procainamide and esmolol were reinitiated in an attempt to gain better control of EAT (Figure 3). The patient’s EAT was well controlled on this antiarrhythmic regimen but given her critically ill status, she was not felt to be a transplant candidate. Family made the decision to withdraw care and she died.

## Discussion

Ivabradine, an inhibitor of the HCN channels, is currently approved for use in adult patients with heart failure and also used in chronic stable angina, inappropriate sinus tachycardia, and postural orthostatic tachycardia syndrome.^4^^,^^9^^,^^10^ It subsequently earned approval for use in pediatric patients with dilated cardiomyopathy following data demonstrating reduction in resting heart rate and improved echocardiographic indexes in this patient population.^1^,^4^ HCN channels, specifically HCN4, expressed in the sinoatrial note help regulate the heart rate via an inward I_*f*_ current owing to sodium and potassium ions entering the cell in response to hyperpolarization. This controls the automatic slow diastolic depolarization of the cells in the sinoatrial node and thus controls heart rate. Inhibition of HCN by ivabradine results in a decreased rate of the slow diastolic depolarization phase and lowers heart rate.^11^

Given its ability to slow heart rate, the use of ivabradine has been reported in various tachydysrhythmias, specifically those due to enhanced automaticity, with promising results. Several case reports and series have shown ivabradine to be beneficial in the management of atrial tachycardia in adolescent and adult patients.^9^^,^^10^^,^^12^ For pediatric patients, ivabradine use has been reported in congenital and postoperative junctional ectopic tachycardia, with success.^3^^,^^4^^,^^13^ Ivabradine has minimal side effects, specifically with regard to hypotension or worsened cardiac function, even in the postoperative population.^4^ Bradycardia is the most frequently reported adverse effect, which may limit use; however, many postoperative patients having pacing wires that can be used to mitigate this effect, as was done in our patient.^5^

EAT, a form of supraventricular tachycardia due to automaticity, can be seen both in pediatric patients with structurally normal hearts and in those with other forms of CHD. Arrhythmias including EAT following surgery for CHD are a concern in the postoperative period, as they can exacerbate an already delicate hemodynamic state with the potential to worsen cardiac output. Risk factors for the development of EAT in the postoperative period following surgery for CHD include lower weight, single-ventricle physiology, longer cardiopulmonary bypass time, The Society of Thoracic Surgeons-European Association for Cardio-Thoracic Surgery (STAT) category, delayed sternal closure, age at surgery, use of milrinone, low serum potassium levels, and atrial trauma.^7^^,^^8^ As demonstrated by our case, EAT can often be an incessant tachycardia; control and management can be challenging, requiring multiple antiarrhythmic agents.

Given the automatic nature of EAT, some authors have reported use of ivabradine for this dysrhythmia in patients with structurally normal hearts.^14^^,^^15^ However, to our knowledge, there are no reports in the literature of the use of ivabradine for EAT in a neonate with single-ventricle CHD. Atrial dilation in the setting of tricuspid regurgitation was the likely aggravating factor in the development of our patient’s EAT. Furthermore, she had heart failure in the setting of volume load on a systemic right ventricle owing to severe tricuspid valve regurgitation. The periods of incessant EAT significantly worsened her already compromised hemodynamics and cardiac output, leading to discussions of the potential need for extracorporeal membrane oxygen support. During these times, manipulation of other antiarrhythmics, including beta blockade and amiodarone, was limited owing to concerns for worsening hypotension. The initiation of ivabradine yielded a useful adjunct to procainamide in this challenging situation, with improvement in EAT and no effect on blood pressure or inotropy in the setting of baseline heart failure. Although there was a period of time the patient’s EAT was controlled on ivabradine alone, it is important to note that this was in conjunction with improved AV valve regurgitation, decreased volume load, and improved hemodynamics secondary to surgery and the patient’s having an open sternum. After chest closure and added hemodynamic stress, the ivabradine alone did not suppress the EAT, but it was able to be used as an adjunct with the procainamide and esmolol infusions. Consideration must be given to the shifts in hemodynamics and the improvement in AV valve regurgitation that took place after surgery, as this clearly impacted the overburden of EAT. This makes it difficult to determine how much of a role the ivabradine actually played in the suppression of the EAT.

Physicians should be aware of chronotropic insufficiency as a result of ivabradine. As infants typically rely on heart rate augmentation to increase their cardiac output owing to limited ability to augment stroke volume, chronotropic insufficiency potentially contributes to insufficient cardiac output in the setting of stress. This was seen in our case; however, this effect of ivabradine can be well managed with atrial pacing to maintain sufficient overall cardiac output. Many postoperative CHD patients have atrial and/or ventricular pacing wires in place, making this a feasible management strategy in this population. We would not advocate for initiation of ivabradine in the patient population of infants under 6 months of age with congenital heart disease without capability for atrial pacing as a backup. Should an infant prove to have acceptable heart rate with no clinical evidence of decreased output on steady-state dosing, ivabradine may be considered for use in the outpatient setting in this patient population.

## Conclusion

Ivabradine has been shown to be useful in arrhythmias due to automaticity. Postoperative infants and children after surgery for CHD are at increased risk for the development of arrhythmia, specifically automatic arrhythmias such as EAT. The currently used antiarrhythmic agents in these situations have numerous adverse effects that can further complicate the clinical scenario, worsening overall hemodynamics. The use of ivabradine to slow the heart rate and control automatic tachycardias with minimal side effects other than bradycardia may prove to be a useful therapeutic modality for these types of patients. The challenge of resultant chronotropic insufficiency can be reasonably mitigated with the use of pacing wires, which are often placed after surgery in these patients.

**Key Teaching Points**

• Ectopic atrial tachycardia is an automatic tachycardia that can be incessant in nature and seen in postoperative pediatric patients. Owing to the tenuous hemodynamic state in the postoperative period, ectopic atrial tachycardia may have more significant hemodynamic consequences.
• Currently, ivabradine is only approved for use in pediatric patients with dilated cardiomyopathy, but numerous studies have shown potential benefit in various forms of automatic tachycardias.
• Ivabradine is the only hyperpolarization-activated cyclic nucleotide-gated (HCN) channel inhibitor currently approved for use in the United States and acts to decrease heart rate with minimal effect on blood pressure or inotropy.
• In neonates, especially with complex congenital heart disease, heart rate increases are vital to augmentation of cardiac output, and thus bradycardia or chronotropic insufficiency can result in clinically significant change in cardiac output.
• As ivabradine acts to decrease heart rate, the clinician must be mindful of the potential for clinically significant decrease in cardiac output with use in a pediatric patient, owing to the effect of bradycardia on cardiac output.
